# Demineralized Bone Matrix Coating Si-Ca-P Ceramic Does Not Improve the Osseointegration of the Scaffold

**DOI:** 10.3390/ma11091580

**Published:** 2018-09-01

**Authors:** Andrés Parrilla-Almansa, Nuria García-Carrillo, Patricia Ros-Tárraga, Carlos M. Martínez, Francisco Martínez-Martínez, Luis Meseguer-Olmo, Piedad N. De Aza

**Affiliations:** 1 Image Diagnostic Service, Virgen de la Arrixaca University Hospital, UCAM-Universidad Catolica San Antonio de Murcia, Guadalupe, 30107 Murcia, Spain; aparrilla10@gmail.com; 2 Preclinical Imaging Unit, Laboratory Animal Service, University of Murcia, 30107 Murcia, Spain; ngc2@um.es; 3 Tissue Regeneration and Repair Group: Orthobiology, Biomaterials and Tissue Engineering, UCAM-San Antonio Catholic University of Murcia, Guadalupe, 30107 Murcia, Spain; p.ros.tarraga@gmail.com; 4 Pathology Unit, IMIB-Arrixaca, 30120 El Palmar (Murcia), Spain; cmmarti@um.es; 5 Orthopaedic and Trauma Service, Virgen de la Arrixaca University Hospital, El Palmar, 30120 Murcia, Spain; fmtnez@gmail.com; 6 Department of Orthopaedic Surgery and Trauma, School of Medicine, Lab of Regeneration and Tissue Repair, UCAM-Universidad Catolica San Antonio de Murcia, Guadalupe, 30107 Murcia, Spain; lmeseguer.doc@gmail.com; 7 Instituto de Bioingeniería, Universidad Miguel Hernández-UMH, Avda. Ferrocarril s/n. Elche, 03202 Alicante, Spain

**Keywords:** dicalcium silicate, tricalcium phosphate, demineralized bone matrix, bone regeneration, micro-CT, histomorphometry

## Abstract

The aim of this study was to manufacture and evaluate the effect of a biphasic calcium silicophosphate (CSP) scaffold ceramic, coated with a natural demineralized bone matrix (DBM), to evaluate the efficiency of this novel ceramic material in bone regeneration. The DBM-coated CSP ceramic was made by coating a CSP scaffold with gel DBM, produced by the partial sintering of different-sized porous granules. These scaffolds were used to reconstruct defects in rabbit tibiae, where CSP scaffolds acted as the control material. Micro-CT and histological analyses were performed to evaluate new bone formation at 1, 3, and 5 months post-surgery. The present research results showed a correlation among the data obtained by micro-CT and the histomorphological results, the gradual disintegration of the biomaterial, and the presence of free scaffold fragments dispersed inside the medullary cavity occupied by hematopoietic bone marrow over the 5-month study period. No difference was found between the DBM-coated and uncoated implants. The new bone tissue inside the implants increased with implantation time. Slightly less new bone formation was observed in the DBM-coated samples, but it was not statistically significant. Both the DBM-coated and the CSP scaffolds gave excellent bone tissue responses and good osteoconductivity.

## 1. Introduction

In order to improve the biomaterials integrated into host bone tissue during bone regenerative engineering, several strategies have emerged. Designing scaffold pore architecture and porosity facilitates more efficient cell and vascular (capillary) irruption. One plausible approach to enhancing bone tissue integration is oxygen and nutrient transport (osteointegration/osseointegration) [1,2,3]. The introduction of cell adhesive molecules has provided promising outcomes for better tissue integration [4]. Tissue integration can also be promoted by designing chemical biomaterial composition by either changing the surface properties of materials or amending the intrinsic chemical structure via coating, patterning, and grafting [3,5]. The use of allografts and xenografts in scaffolds as a bone tissue substitute is another major research area given the described complications and morbidity attributable to autologous graft procedures, and the limitation of availability/stock when required in large quantities [6]. Another point raised in recent studies has been the addition of osteoinductive bioactive components as they lead to greater scaffold osseointegration, and prevent its encapsulation and loss [7].

Demineralized bone matrix (DBM) is an allogeneic bone graft from bone that has been acid-treated to remove the inorganic mineral fraction while maintaining the organic collagen matrix (93%). The DBM also contains 5% of the full array of naturally occurring growth factors that provide the osteoinductive protein signal to form new bone (transforming growth factors (TGFs), insulin growth factor (IGF), fibroblast growth factor (FGF), and bone morphogenetic protein (BMP)) [8,9]. DBM products are available in several commercial presentations, such as morselized material, powder, putty pastes, chips, porous foams, gel, etc. [10,11,12]. Nevertheless, a high osteogenic capacity rate of bone is lost while it is processed [13,14]. As a result of the demineralization process, the mechanical properties (given by the inorganic matrix) significantly diminish, and the DBM is biologically more active than a standard mineralized allograft. DBM has been clinically used since the beginning of the 1980s [15], but evidence for or against its ability to form new bone is still poor as osteogenic activity is age- and gender-related, and depends on the lifestyle habits (drinking alcohol, smoking habit, etc.) of both the donors and the carrier or excipient used to prepare it.

Calcium-phosphate-based material in the apatite phase is the mineral fraction of bone tissue. For this reason, calcium phosphate ceramics, classically tricalcium phosphate (TCP) and hydroxyapatite (HA) ceramics, are generally used for osseous repairs and regeneration given their biocompatibility and comparable biochemical composition with living apatite in bone [16]. In the calcium phosphate materials that contain silicon, the role of Si in the growth of healthy bone and connective tissues is recognized [17,18,19]. Silicon also modifies material properties and increases the in vivo properties of calcium phosphate materials that contain silicon. Consequently, monophasic or biphasic calcium silicophosphate (CSP) materials have been extensively studied as biomaterials per se or have been accompanied with cells (scaffolds) for bone tissue replacement. More attention is being paid to the CSP biomaterial for its good bioactivity response and low cytotoxicity [17,18,19].

It is believed that adding DBM to ceramics improves stability, encourages new bone formation, and confers better structural properties. Accordingly, this study aimed to manufacture and characterize CSP ceramic (control) and DBM-coated CSP ceramic as promising candidates for the preparation of bioceramic scaffolds with enhanced osteopromotion capacity, which implies improved osteoinductive properties, and to compare their physical and in vivo behavior. The novelty of this study lies in the scaffold’s composition and coating with a DBM layer on a CSP-based ceramic to evaluate in vivo behavior in order to find a new potential use of the DBM as an additive to the ceramic or inorganic materials used in bone tissue engineering.

## 2. Materials and Methods

### 2.1. Scaffold Preparation and Characterization

Laboratory solid-state sintered γ-dicalcium silicate and α-tricalcium phosphate were utilized as raw materials [20]. The stoichiometric quantities of the starting powders (dicalcium silicate (45 wt %)/tricalcium phosphate (55 wt %)) were attrition milled using Y-PZS balls in isopropyl alcohol medium (45 min), dried (60 °C overnight), isostatically pressed (200 MPa), and heated inside platinum crucibles to 1000 °C for 2 h at a rate of 10 °C min^−1^. To ensure homogeneity, bars were ground, pressed isostatically, and reheated to 1300 °C for 24 h. This procedure was repeated twice. One part of the obtained biphasic material was ground inside a tungsten carbide mill to the 1–2 mm particle size. The other part was milled in a planetary grinder to a mean 2 µm particle size, according to laser scattering (Master Sizer S; Malvern Instruments Limited, Worcestershire, UK).

**Figure 1:**
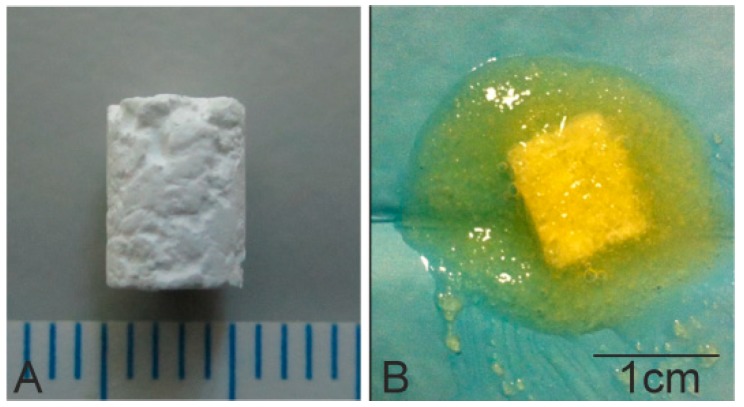
Photographs of the (**A**) calcium silicophosphate (CSP) scaffold and (**B**) the demineralized bone matrix (DBM)-coated scaffold.

Scaffold implants were prepared with 10% of 2 µm powder and a 90% coarse fraction powder of 2–1 mm by adding 10% polyvinyl acetate (PVA) as a binder. The biomaterial was then heated to 1170 °C for 2 h before being allowed to cool for 24 h inside the furnace. Scaffolds were then cut into 6 mm long cylinders with diameter 4.5 mm (Figure 1A).

Afterward, scaffolds were placed inside individual containers and were sterilized by hydrogen peroxide gas plasma (Sterrad^®^ 100S, Johnson&Johnson, Irvin, CA, USA) at low temperature.

Subsequently, gel DBM (DBM-Osteoplant Angiostad^®^, Bioteck, Arcugnano, Italy) was used to coat the previously manufactured scaffolds. Scaffolds were immersed in 231.3 ± 1.35 mg of gel DBM for 3 min, and were then weighed on a balance; the amount of DBM adhered to the scaffold surface via the surface pores was 79.06 ± 0.47 mg (Figure 1B). The macroscopic morphology structure of the scaffolds was realized using a Nikon D3400-DSLR (Nikon, Tokyo, Japan).

The scaffolds’ microstructure was characterized by Scanning Electron Microscopy fitted with Energy-Dispersive X-ray Spectroscopy (SEM-EDX, Ibaraki, Japan), SEM-Hitachi S-3500N and INCA system, by Oxford Instruments Analytical, UK, respectively). Samples were palladium-covered according to a previous literature protocol [21]. The crystalline phases present in the scaffolds was identified in an AXS D8Advance XRD (Bruker, Karksruhe, Germany).

Finally, in order to acquire information about the major functional groups of the scaffolds, Fourier Transform Infrared Spectrometry (FTIR Thermo SCIENTIFIC Nicolet iS5, equipped with an iD5 ATR Accessory) was used. Data were collected between 4000 and 550 cm^−1^ at 20 °C and at 64 scan resolution. Gel DBM was also used for comparison purposes with both scaffolds.

### 2.2. Animal Protocol 

The study protocol was approved by the Institutional Ethic and Animal Experimentation Committee (Murcia University) according to Spanish Government and European Community Animal Care Guidelines (authorization no. A13150102).

Twenty-one male New Zealand pathogen-free rabbits, whose initial weight was 4–4.5 kg, aged 26–28 weeks-old (skeletally mature to ensure growth plates or physes closure) with one tibial bone defect per animal (5 mm in diameter × 6 mm long), were included in this study. Animals were allocated randomly to three groups (*n* = 7 each), respectively corresponding to three study periods of 1, 3, and 5 months. In each group, the biomaterial implant was doped, or not, with gel DBM, plus a similar bony defect (5 mm in diameter) with no biomaterial implanted as the control defect.

The surfaces of nine synthesized pieces (*n* = 3 per testing period) were coated with gel DBM and nine others (*n* = 3 per testing period) were used with no coating, plus three controls, one for each implantation time. The surgical procedures were performed under general anesthesia and sacrificing procedures were as carried out previously by our group [17]. In a brief, the anesthetic method was administration of atropine sulfate (0.3 mg kg^−1^, via i.m.); chlorpromazine hydrochloride (10 mg kg^−1^, via i.m.); Xylacine (0.25 mg kg^−1^, via i.m.); and Ketamine hydrochloride (50 mg kg^−1^, via i.m.). Then, animals were given a broad-spectrum antibiotic prophylaxis (Enrofloxacin at 15 mg kg^−1^, via i.m., single dose). Postoperative pain was controlled with mepivacaine 1% via subcutaneous applied in the surgical wound and buprenorphine (0.3 mg kg^−1^, via i.m., b.i.d.—two time a day—for 4 days).

To achieve a standardized or uniform procedure, all the interventions were performed by the same surgeon (L. Meseger-Olmo) and also in order to avoid variations in the place where the cortical osseous defect was carried out, the anterior tibial tuberosity was used as an anatomical landmark. To perform the bony defect, the bone surface was approached by means of a small straight anteromedial direct incision (approx. 1.5–2 mm), taking as anatomical references the anterior relief of tibial tuberosity and parallel to the shaft axis (midway between the anterior and posterior edge). After exposing it, we performed a careful periosteal separation to present the cortical surface. A bone unicortical defect was performed using a 5.0 mm diameter surgical drill bit coupled to a micromotor at low revolutions (1000 rpm) and continuous cooling by copious irrigation with physiological saline solution without invading the medullary cavity, creating a contained bone defect that facilitated the stability of the implanted material in the osseous receptor bed. Then, the defect was rinsed with physiological saline solution to remove remaining bone debris and chips produced during the realization of the defect. Some defects were directly grafted with DBM-coated scaffold and CSP scaffold, while others remained empty.

### 2.3. Histological and Histomorphometric Analyses

**Figure 2:**
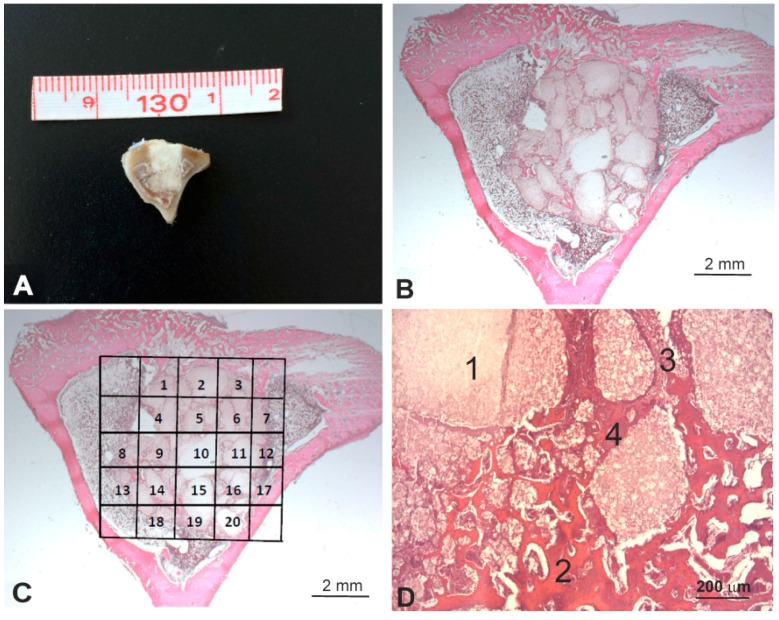
(**A**) Representative image of the macroscopic appearance of the implanted scaffold, (**B**) microscopic appearance of the implant stained with hematoxylin and eosin, (**C**) manually traced individual regions of interest (ROIs) by including only the scaffold, and (**D**) the four different components analyzed in the histomorphometric analysis (1, residual scaffold; 2, neoformed bone; 3, connective tissue; and 4, fibroblastic tissue). Hematoxylin and eosin stain (**B**–**D**).

After euthanasia at the predetermined time points (1, 3, and 5 months), the entire tibia was excised and a bony segment containing the original bone defect site with the scaffold, along with some surrounding tissue, was extracted and processed for the histological observations and the histomorphometric evaluation (Figure 2A). Samples were fixed in 4% buffered formalin (Panreac Química, Barcelona, Spain) for 24 h, and were decalcified for 7 days using a formic-acid-based 21–23% solution (Shandon TBD2, Thermo Corp., Madrid, Spain). Samples were then dehydrated, processed, and paraffin-embedded. Four-micrometer transversal sections, laid perpendicularly to the bone axis, were obtained at three different levels (one section per 100 micrometers), and were stained according to a standard hematoxylin and eosin stain protocol (Figure 2B).

The histomorphometric evaluations were made after manually tracing the individual regions of interest (ROIs) by including only the scaffold (Figure 2C). To assess the effect of the influence of the gel DBM coating on implant behavior, four different items were measured (Figure 2D): residual implant, newly formed bone, neoformed connective tissue, and neoformed fibroblastic tissue (the basophil and sandy deposits located next to the neoformed bone tissue). The surface of every item was determined in each microphotograph. The total implant surface of each item was determined by the sum of all the data in the microphotographs from all the sections.

Examinations were performed under a Zeiss Axio Scope A.1 bright field optical microscope (Carl Zeiss, Jenna, Germany) connected to a digital camera (Axio Cam IcC3, Carl Zeiss, Jenna, Germany), supplied by IMIB-Arrixaca Pathology Platform. After calibrating the system and digitalizing the images, the interactive measurements of the ROIs were taken with AxioVision Rel. 4.8, Zeiss image analysis software (Carl Zeiss, Jenna, Germany).

### 2.4. Micro-CT Evaluation 

The excised proximal segments of the tibiae that contained a scaffold were micro-CT scanned using a trimodal Albira preclinical PET/SPECT/CT system for small animal imaging (Bruker^®^ Corporation, Karksruhe, Germany) (source voltage 45 kV, source current 0.2 mA, axial slices 0.5 mm). A digital flat panel X-ray detector (Bruker^®^, Karksruhe, Germany), with 2400 × 2400 pixels and a 70 × 70 mm^2^ field of view, was used to capture 1000 projections of 0.05 mm voxels per sample and the scaffold implantation time.

The images of the CSP scaffolds and DBM-coated CSP prior to being implanted (controls), and the images of tibiae containing the scaffolds, were reconstructed on the three spatial planes by the filtered backprojection algorithm (FBP). Bone mineral density was quantified around the implant in Hounsfield units (HU) using a medical image data examiner (AMIDE, UCLA University, LA, USA), with 3D reconstruction using the Volview image analysis software (http://kitware.com/opensource/volview.html, Kitware, Clifton Park, NY, USA). A mathematical study of the raw data was conducted in the Biostatistics Department (UCAM (Catholic University of Murcia, Guadalupe, Spain)).

**Figure 3:**
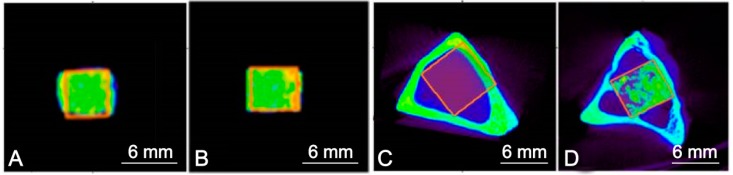
ROIs of (**A**) the CSP scaffold, (**B**) the DBM-coated CSP, (**C**) the rabbit tibiae before implantation, and (**D**) the scaffold after implantation as being representative of the two implanted materials.

An entire rectangle of each section was traced manually to create individual ROIs. The ROIs of the controls contained all the scaffolds, the gel DBM, and the rabbit tibiae, while the ROIs after implantation contained bone, implant particles, and bone/connective tissue (Figure 3).

### 2.5. Statistical Analysis

Histomorphometric data: statistical analysis was performed with the GraphPad Prism 7.0^®^ commercial software (GraphPad Software, version 7.0, San Diego, CA, USA). All the values are reflected by their respective standard errors (SEs) of the mean. To establish differences between the measured parameters, a Mann-Whitney nonparametric test was applied based on lack of data normality according to the D’Agostino and Pearson normality test. The results were statistically significant for a *p* value of <0.05.

The micro-CT data: a linear regression analysis was performed using R Statistical Software (Foundation for Statistical Computing, Vienna, Austria), a free software environment for statistical computing and graphics. For the relationships between the values of each volume of interest, an overall analysis of all the statistical values was performed, including median, principal components, variability, standard deviation, minimum value, maximum value, ROI size in mm^3^, fraction voxels, and total voxels.

## 3. Results

**Figure 4:**
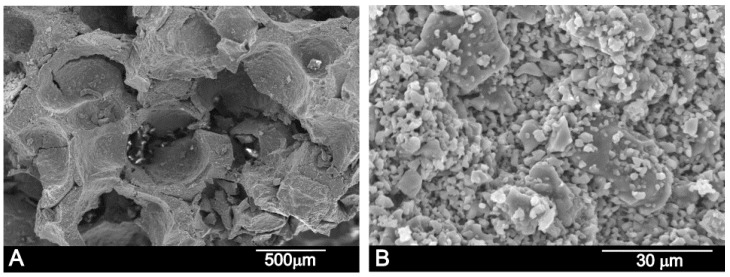
(**A**) SEM and (**B**) high-resolution SEM images of the manufactured CSP scaffold.

Figure 4 shows the scaffold’s microstructure. The structure of the manufactured scaffold presents large porosity which, according to the SEM observations, displayed pore diameters that fluctuated from 250 to 500 µm. The scaffold was obtained by the partial sintering of different-sized porous granules, which were observed in the high-magnification SEM image (Figure 4A,B). On the other hand, micropores from 2 to 5 µm were also visualized in between the particles (Figure 4B). The apparent density was 1.6 g cm^−3^, which meant that total porosity was 57%.

Scaffold composition was established with a quantitative EDS analysis at several different sample points, and was around 57.0 ± 2.0 wt % CaO, 27.7 ± 5.5 wt % P_2_O_5_, and 15.3 ± 5.5 wt % SiO_2_—close to the composition of the synthetized material determined by chemical analysis (56 wt % CaO, 24 wt % P_2_O_5_, 13 wt % SiO_2_).

**Figure 5:**
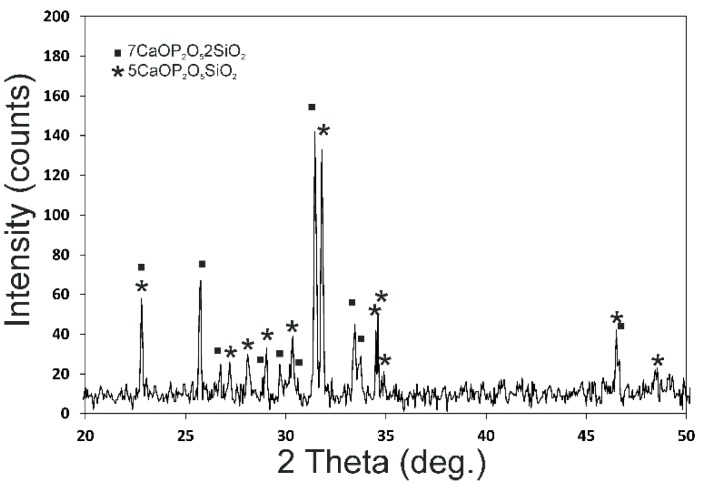
X-ray diffraction pattern of the synthesized CSP scaffold.

Figure 5 shows the X-ray diffraction patterns of the CSP scaffold. The sharp peaks and low backgrounds suggested that the powder was highly crystalline. The CSP scaffold presented a mixture of Nurse’s A (7CaOP_2_O_5_2SiO_2_-JCPDS card No. 011-0676) and silicocarnotite (5CaOP_2_O_5_SiO_2_-JCPDS card No. 40-393) phases according to the subsystem Nurse’s A-phase–silicocarnotite within the system Ca_3_(PO_4_)_2_-Ca_2_SiO_4_ [22].

**Figure 6:**
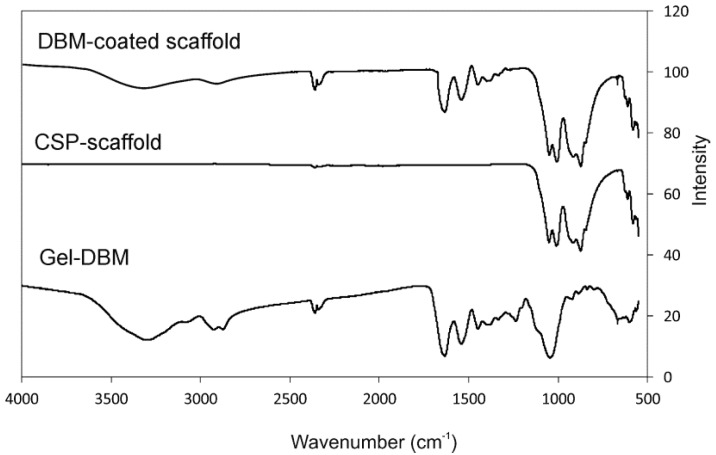
The FTIR of the CSP scaffold, the DBM-coated scaffold, and gel DBM for comparison purposes.

Figure 6 shows the FTIR spectra of both the coated and uncoated scaffolds, as well as the gel DBM, for comparison purposes. The CSP scaffold presents the bands brought about by the SiO_2_ and PO_4_^2−^ groups. The DBM-coated scaffold also shows gel DBM bands.

The IR bands groups in the 550 to 1100 cm^−1^ spectral region are shown in the scaffolds. A first group is observed between 550 and 650 cm^−1^, with a second group between 800 and 1100 cm^−1^.

The different dicalcium silicate polymorphs present FTIR bands at around 850, 875, 895, 925, 995, and 1010 cm^−1^, which corresponds to ν_1_ (symmetric stretching) and ν_3_ (antisymmetric stretching). The different polymorphs of the calcium phosphate materials show FTIR bands, triply degenerated (ν_4_) between 550 and 620 cm^−1^, the P-O stretching mode (ν_3_) between 1125 and 1070 cm^−1^, the doubly degenerated O-P-O bending mode (ν_2_) between the bands at 420 and 460 cm^−1^, and the nondegenerated symmetric terminal P-O stretching bands (ν_1_) at 970 cm^−1^, all based on the bands of the phosphate and silicate species reported in the bibliography [23,24].

The FTIR bands of the CSP scaffold present peaks at 562, 581, 611, 626, 846, 874, 917.25, 1009, and 1050 cm^−1^, attributed to the phosphate and silicate units of its structure. The FTIR bands of the DBM-coated scaffold present SiO_2_ and PO_4_^2−^ groups and collagen peaks. Above 1100 cm^−1^, all the peaks came from collagen vibrations, except for the band at 3400 cm^−1^, which was attributed to the structural OH- groups, and the 1460 cm^−1^ peak, attributed to the CO_3_^2−^ group [25,26].

**Figure 7:**
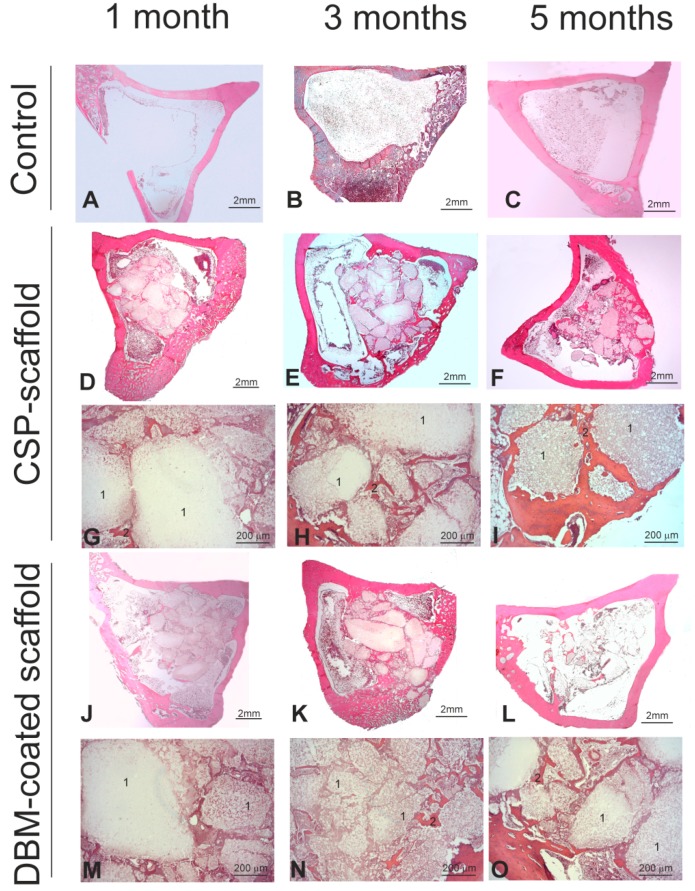
(**A**–**C**) Representative images of the tibia samples from the control, (**D**–**I**) implanted CSP and (**J**–**O**) DBM-coated scaffolds. In the controls, the bone defect (**A**) was repaired by physiological bone repair at months 3 and 5 (**B**,**C**, respectively). In the CSP group, the scaffold surface progressively decreased (**D**–**I**, number 1) with increasing neoformed bone organization (**G**–**I**, number 2). Similarly, the DBM-coated scaffold surface progressively decreased from month 1 to month 5 (**J**,**K**,**M**–**O**, number 1) but, in this case, the neoformed bone organization slightly diminished (**M**,**N**, number 2) compared with the CSP scaffold. Hematoxylin and eosin stain. (1, residual scaffold; 2, neoformed bone).

Figure 7 shows the histological results of the CSP scaffold, the DBM-coated scaffold, and the control defect implanted at 1, 3, and 5 months. All the animals survived the previously established time points. In macroscopic terms, no evident signs of either inflammation or infection at the implantation sites were observed at any point.

Histologically speaking, the control cortical of the unfilled bone defect (Figure 7A), similar to that performed to implant the materials, was cured spontaneously following the physiological repair and remodeling process by closure with cancellous bone tissue at 3 and 5 months (Figure 7B,C). When the CPS scaffold was implanted, a progressive decrease of the scaffold from month 1 to month 5 was generally observed (Figure 7D–F). The resorption pattern of the scaffold samples indicated many resorption foci inside and on scaffold surfaces, with an irregular pattern. Apparently, augmented bone formation took place inside the medullar zone, with the neoformed bone tissue surrounding the remaining scaffold particles (Figure 7G–I). This finding confirmed its osteoconductive property. For the DBM-coated scaffold, the degree of resorption of the whole implant was similar to that described for the CPS scaffold (Figure 7J–L), but the new bone formation seemed slightly lower (Figure 7M–O).

The histomorphometric quantification confirmed the histopathological analysis (Table 1). Although there were several statistical differences between the different parameters measured at months 1 and 3, compared mainly with those at 5 months post-surgery, no significant differences were observed between these parameters in both groups at month 5 after the scaffold implant.

**Figure 8:**
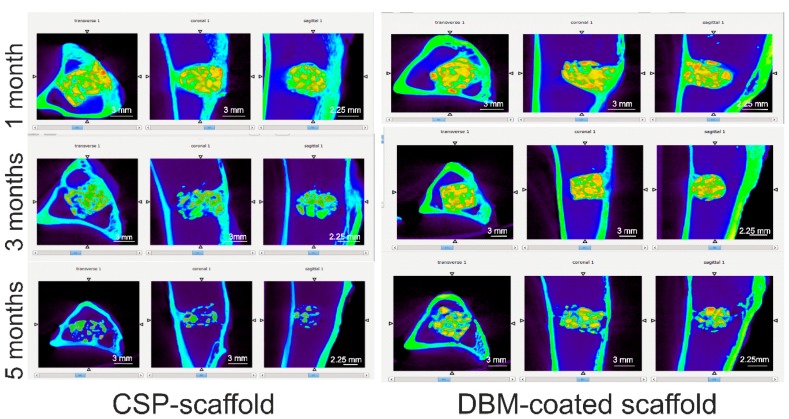
The micro-CT images of both materials implanted in tibiae at 1, 3, and 5 months post-surgery. Orange color corresponds to residual biomaterial. Yellow color corresponds to biomaterial partially reabsorbed. Green color corresponds to the cortical bone and represents the normal cortex of the host bone. Green color located within the biomaterial represents the reabsorbed biomaterial and new bone tissue. Blue color corresponds normal medullary tissue of the host bone. Blue color located within the biomaterial represents neoformed fibrous connective tissue.

Figure 8 summarizes the micro-CT images of the CSP scaffold and the DBM-coated scaffold in the transversal, coronal, and sagittal views after 1, 3, and 5 months of implantation in rabbit tibiae. The micro-CT images show that both scaffolds are well positioned in the bony defects.

We observe that as the implantation time is prolonged, the volume of both scaffolds decreases. This implies the degradation of both ceramic materials, which was more pronounced in the CSP scaffold after 5 months than in the DBM-coated scaffold. The tissue inside the scaffolds and around them had grown.

**Figure 9:**
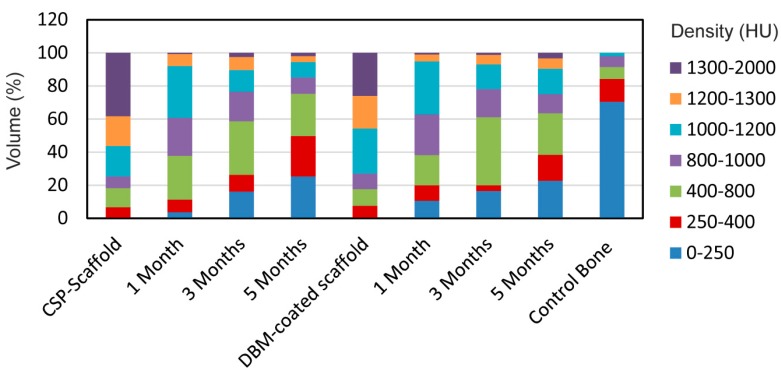
The density regions displayed as a percentage of the total volume of the ROIs after implantation months 1, 3, and 5, and in control, CSP scaffold, and DBM-coated scaffold bone specimens with densities given in HU.

Figure 9 shows the density distribution of the scaffolds in HU, performed to determine how similar the compositions of the implanted scaffolds were to the control scaffolds (original scaffold before implantation), and also to the control bone tissue section. So, if both scaffolds increased the density within the entire bone tissue density range (0–1000 HU), this would mean that the scaffolds would have been reabsorbed by the surrounding tissue and transformed into new tissue. If, however, the scaffolds did not change during the implantation time, then a high density range would remain (1200–2000 HU).

The results showed that both scaffold groups had a majority of material density that fell within the 1000–2000 HU range, with an almost opposite distribution observed in the control bone, and 70% of the densities between 0 and 250 HU making up the control bone volume. Both implanted scaffolds showed an increased distribution in densities, but less than those found in the control scaffolds. While this displays that the scaffold groups were dissimilar to the control bone in density distribution terms, the reduced density, compared with the control scaffold, revealed that remodeling occurred throughout the scaffold volume as implantation time was prolonged.

## 4. Discussion

A newly developed biphasic calcium silicophosphate scaffold ceramic, coated with a natural demineralized bone matrix, was tested herein for its bone healing capacity and osteoinductivity by comparing its behavior with that of a pure CSP scaffold. A full quick reconstruction of bone defects through the bone-related tissue of autologous/allogeneic bone grafts is essential in trauma, maxillofacial, and orthopedic surgery. Calcium phosphate scaffolds in bone tissue engineering should be designed to mimic extracellular bone matrix properties and structure, and the present work intended to generate and test scaffolds with similar physico-biological properties to those of natural bone tissue [17].

By the partial sintering of different-sized porous granules, we prepared a highly interconnected porous scaffold within the 250–500 µm range that met bone tissue in-growth requirements, and also transported the essential nutrient for cell survival and proliferation. We also coated the scaffold with a natural DBM to check whether this coating improved the CSP scaffold’s material properties. The main discoveries were that both ceramics degraded over the experimental set points, and induced bone formation inside and around the scaffold, which was already observed during the first study period by the histology, histomorphometric, and micro-CT analyses and evaluations.

Both the interfaces generated at the biomaterial and the new bone tissue were studied by numerous techniques. The new bone structure was characterized differently depending on the selected examination technique: light microscopy or electron microscopy (SEM and TEM) [27,28,29] and the traditional method of histologically staining thin sections, followed by light microscopy examination, which confers considerable information but of an ultrastructural kind. The consistency and exactness of a histomorphometric study into newly formed bone depend on the proper identification and ultrastructural characterization of all the biological constituents that might form part of the osseointegration process [1,19].

Our histological and histomorphometric results showed biomaterial disintegration and the presence of isolated ceramic fragments dispersed in the medullary cavity over the 5-month study period. After 3 months, the implants had lost their boundaries, displayed increased porosity, and had begun a neo-trabeculation process. After 5 months, the implants had gradually disintegrated and the presence of free ceramic fragments dispersed inside the medullary cavity became more evident. No difference was found between the DBM-coated and the uncoated implants. The new bone tissue inside the implants increased with implantation time. Slightly less new bone formation was found in the DBM-coated samples, but it was not statistically significant. In relation to the connective tissue (granulation tissue) produced inside the implants, an increase was noted until month 3, followed by a sharp drop in both implanted materials by month 5. This granulation tissue can be considered a precursor to new bone formation. Thus, diminished connective tissue overlapped the increase in new woven bone and mature bone in both materials.

To date, very few studies have added DBM inorganic materials. Generally, DBM is accompanied by growth factors or mesenchymal stem cells [9,12,13,14]. The addition of natural demineralized or decellularized materials to colloidal gels had surprising effects in bone tissue restoration terms. Typically, addition of DBM did not increase the regenerative potential compared with HA-HAp hydrogels, whereas adding decellularized cartilage produced, on average, superior bone tissue restoration compared with DBM gels [30]. Other studies which have compared the DBM collagen structure prepared with three different particle sizes have concluded that medium particle sizes (0.5–1 mm) of DBM in vivo are desirable when the DBM is implanted alone, while small particles (<0.5 mm) are recommended when the DBM is implanted together with mesenchymal stem cells. Moreover, when comparing both groups, the neoformed bone tissue around the DBM with the mesenchymal stem cells is much higher than around the DBM alone [10].

Our results are similar to those in other studies in which DBM was implanted together with calcium phosphate cement, where the calcium phosphate cement acted by inhibiting osteogenesis when associated with a DBM [11].

Further micro-CT examinations shed more light on the bone formation and implant resorption processes, and micro-CT proved to be the best radiographical method to qualitatively and morphologically study bone structure and density. It is an excellent device for measuring the relative distribution of compact and cancellous bone tissue on a bone density scale. Table 2 shows the distinction among the four bone types in HU units [31,32,33,34]. The Hounsfield scale was used to evaluate bone densities for implant placement, and the results were considered site-specific, objective, and quantitative. Engineers, odontologists, and maxillofacial and orthopedic surgeons use HU values to design custom oral, facial, and skeletal implants [35,36,37]. Numerous reports have considered bone density an important factor for the long-term success of osseointegrated implants [38,39,40].

Our micro-CT scan results drew similar conclusions to our histological and histomorphometric studies, and observed a decreasing tendency for the volume occupied with wide-ranging densities, which corresponded to the unintegrated scaffold (1200–2000 HU), and a favored increase in the value of tissue with narrow-ranging densities (0–1200 HU). No statistical differences were observed between the CSP scaffold and the DBM-coated scaffold by the end of the study.

Before implantation, more than 75% of the material density volume obtained high HU values, between 1000–2000 HU, and more than half of that percentage corresponded to ranges wider than 1200 HU, which are related to implant type. The high-density ceramic materials (the scaffold was produced at high temperature, 1300 °C for 24 h) had high HU values. The presence of ~25% of the volume with a low HU (250–1000 HU) was due to the heterogeneous constitution of the scaffolds (i.e., presence of pores).

At 1 month, hardly any volume was occupied by the 0–250 HU values as both scaffolds were implanted in the medullary cavity area, which came into contact with the cortex. With time, volumes decreased with wider ranges than 1000 HU, which meant that the implants had degraded and transformed. During month 3, the volumes with densities between 400 and 800 HU predominated and the tissue with a density lower than 250 HU significantly increased. By the end of the study, more than 85% of the volume was below 1000 HU for the CSP scaffold, with 75% of the volume for the DBM-coated scaffold.

In spite of the New Zealand rabbit’s swift metabolic activity, its validity as an experimental model to test the biomaterials employed in bone replacement has been proven in former studies [1,7,26,27,28].

Our future research will test the addition of osteoinductive factors, such as morphogenetic proteins, which may speed up the resorption rate and stimulate bone formation at the same time. We will also compare DBM with standard calcium phosphate materials, such as HA or TCP, in an attempt to establish the new potential use of DBM as an additive to ceramic materials used in bone tissue engineering.

## 5. Conclusions

The comparison of the micro-CT scan and histological images demonstrates that the new tissue between the remaining ceramic fragments (the hypodense material between the hyperdense biomaterial shown in the micro-CT images according to HU) is identified histologically as being osteogenic and connective tissue (granulation tissue). This finding enables a correlation to be made among the visual appearance in 3D, the raw data, and the tissue type present in the histological images.

However, no statistically significant difference was observed in the in vivo behavior of the CSP scaffold and the DBM-coated scaffold. Both materials are biodegradable, bioactive, and present osteoinductive and osteoconductive properties.

## Figures and Tables

**Table 1:** Histomorphometric analysis for the biphasic scaffold (micrometers^2^).

Items	Implantation Time								
	CSP Scaffold			DBM-Coated Scaffold			Control		
	1 month	3 months	5 months	1 month	3 months	5 months	1 month	3 months	5 months
	(a)	(b)	(c)	(d)	(e)	(f)			
Total Scaffold Surface	27.98 ± 1.84	23.07 ± 0.32	16.39 ± 1.93	27.29 ± 1.46	24.80 ± 1.13	16.62 ± 1.52	0.00 ± 0.0	0.00 ± 0.0	0.00 ± 0.0
	** (b,c,f)	** (c,f)		** (c,f)	** (c,f)				
Scaffold Residual	23.75 ± 1.73	18.32 ± 0.40	11.72 ± 2.10	23.28 ± 1.66	19.88 ± 1.10	11.99 ± 1.53			
	** (b,c,f)	** (c,f)		** (c,f)	** (c,f)				
New bone tissue	1.34 ± 0.20	2.21 ± 0.14	3.85 ± 0.17	1.14 ± 0.23	2.21 ± 0.30	3.29 ± 0.14	2.32 ± 2.12	4.14 ± 1.02	5.32 ± 3.13
	* (b,e), ** (c,f)	** (c,f)		* (b,e), ** (c,f)	* (c)				
Connective tissue	1.14 ± 0.32	1.67 ± 0.40	0.43 ± 0.23	1.28 ± 0.10	1.83 ± 0.25	0.82 ± 0.10			
				* (c)	** (c,f)				
Fibroblastic tissue	1.75 ± 0.34	0.87 ± 0.11	0.39 ± 0.12	1.59 ± 0.21	0.88 ± 0.10	0.52 ± 0.18			
	* (c,f)	* (c)		* (b,e,f), ** (c)	* (c)				

Nonparametric Mann–Whitney test. Mean ± SE. Significant differences * *p* < 0.05 or ** *p* < 0.005 among implantation times (a, b, c, d, e, f).

**Table 2:** Norton [32] classification based on the micro-CT scan determinations of bone density.

Micro-CT Value (HU)	Type of Bone
1000–1200	Homogeneous and compact bone
800–1000	Dense cortical-spongy bone
400–800	High-density cortical-spongy bone
250–400	Low-density cortical-spongy bone
0–250	Very soft bone with incomplete mineralization

## References

[1] Johansson P., Jimbo R., Kozai Y., Sakurai T., Kjellin P., Currie F., Wennerberg A. (2015). Nanosized Hydroxyapatite Coating on PEEK Implants Enhances Early Bone Formation: A Histological and Three-Dimensional Investigation in Rabbit Bone. Materials.

[2] Mate-Sanchez de Val J.E., Mazon P., Calvo-Guirado J.L., Delgado-Ruiz R.A., Ramirez-Fernandez M.P., Negri B., Abboud M., De Aza P.N. (2014). Comparison of three hydroxyapatite/β-tricalcium phosphate/collagen ceramic scaffolds: An in vivo study. J. Biomed. Mater. Res. A.

[3] Neacsu P., Staras A.I., Voicu S.I., Ionascu I., Soare T., Uzun S., Cojocaru V.D., Andreea Pandele M., Croitoru S.M., Miculescu F. (2017). Characterization and In Vitro and In Vivo Assessment of a Novel Cellulose Acetate-Coated Mg-Based Alloy for Orthopedic Applications. Materials.

[4] Rabadan-Ros R., Velásquez P., Meseguer-Olmo L., De Aza P.N. (2016). Morphological and structural study of a novel porous Nurse´s A ceramic with osteoconductive properties for tissue engineering. Materials.

[5] Fernandez-Pradas J.M., Serra P., Morenza J.M., De Aza P.N. (2002). Pulser laser deposition of pseudowollastonite coatings. Biomaterials.

[6] Ramirez-Fernandez M.P., Mazon P., Gehrke S.A., Calvo-Guirado J.L., De Aza P.N. (2017). Comparison of two xenograft materials used in sinus lift procedures: Material characterization and in vivo behavior. Materials.

[7] Calvo-Guirado J.L., Ramirez-Fernandez M.P., Delgado-Ruiz R.A., Mate-Sanchez de Val J.E., Velasquez P., De Aza P.N. (2014). Influence of biphasic α-TCP with and without the use of collagen membranes on bone healing of surgically critical size defects. A radiological, histological and histomorphometric study. Clin. Oral Implants Res..

[8] Murata M. (2005). Bone engineering using human demineralized dentin matrix and recombinant human BMP-2. J. Hard Tissue Biol..

[9] Kim Y.K., Um I.W., An H.J., Kim K.W., Hong K.S., Murata M. (2014). Effects of demineralized dentin matrix used as an rhBMP-2 carrier for bone regeneration. J. Hard Tissue Biol..

[10] Dozza B., Lesci I.G., Duchi S., Della Bella E., Martini L., Salamanna F., Falconi M., Cinotti S., Fini M., Lucarelli E. (2017). When size matters: Differences in demineralized bone matrix particles affect collagen structure, mesenchymal stem cell behavior, and osteogenic potential. J. Biomed. Mater. Res. Part A.

[11] Ferreira da Silva L.C., Granja Porto G., Sávio de Souza Andrade E., Rodrigues Laureano Filho J. (2018). Demineralized bone matrix and calcium-phosphate cement in bone regeneration in rats. Acta Cir. Bras..

[12] Kim S.Y., Kim Y.K., Park Y.H., Park J.C., Ku J.K., Um I.W., Kim J.Y. (2017). Evaluation of the healing potential of demineralized dentin matrix fixed with recombinant human bone. Morphogenetic protein-2 in bone grafts. Materials.

[13] Li J., Yang J., Zhong X., He F., Wu X., Shen G. (2013). Demineralized dentin matrix composite collagen material for bone tissue regeneration. J. Biomater. Sci. Polym. Ed..

[14] Wang J.C., Alanay A., Mark D. (2007). A comparison of commercially available demineralized bone matrix for spinal fusion. Eur. Spine J..

[15] Edgar L., McNamara K., Wong T., Tamburrini R., Katari R., Orlando G. (2016). Heterogeneity of Scaffold Biomaterials in Tissue Engineering. Materials.

[16] Canillas M., Pena P., Aza A.H., Rodríguez M.A. (2017). Calcium phosphates for biomedical applications. Bol. Soc. Esp. Ceram. Vidr..

[17] Ros-Tarraga P., Mazon P., Rodriguez M.A., Meseguer-Olmo L., De Aza P.N. (2016). Novel resorbable and osteoconductive calcium silicophosphate scaffold induced bone formation. Materials.

[18] Velasquez P., Luklinska Z.B., Meseguer-Olmo L., Mate-Sanchez de Val J.E., Delgado-Ruiz R.A., Calvo-Guirado J.L., Ramirez-Fernandez M.P., De Aza P.N. (2013). αTCP ceramic doped with Dicalcium Silicate for bone regeneration applications prepared by powder metallurgy method. In vitro and in vivo studies. J. Biomed. Mater. Res. A.

[19] Mate-Sanchez de Val J.E., Calvo-Guirado J.L., Delgado-Ruiz R.A., Ramirez-Fernandez M.P., Martinez I.M., Granero-Marin J.M., Negri B., Chiva-Garcia F., Martinez-Gonzalez J.M., De Aza P.N. (2012). New block graft of α-TCP with silicon in critical size defects in rabbits: Chemical characterization, histological, histomorphometric and micro-CT study. Ceram. Int..

[20] Rubio V., de la Casa-Lillo M.A., De Aza S., De Aza P.N. (2011). The system Ca_3_(PO_4_)_2_–Ca_2_SiO_4_: The sub-system Ca_2_SiO_4_-7CaOP_2_O_5_2SiO_2_. J. Am. Ceram. Soc..

[21] Martinez I.M., Meseguer-Olmo L., Bernabeu-Esclapez A., Velasquez P.A., De Aza P.N. (2012). In vitro behavior of α-Tricalcium Phosphate doped with Dicalcium Silicate in the system Ca_2_SiO_4_–Ca_3_(PO4)_2_. Mater. Charact..

[22] Ros-Tarraga P., Mazon P., Meseguer-Olmo L., De Aza P.N. (2016). Revising the Subsystem Nurse’s A-Phase-Silicocarnotite within the System Ca_3_(PO_4_)_2_-Ca_2_SiO_4_. Materials.

[23] Antonakos A., Liarokapis E., Leventouri T. (2007). Micro-Raman and FTIR studies of synthetic and natural apatites. Biomaterials.

[24] Lugo G.J., Mazón P., De Aza P.N. (2015). Phase transitions in single phase Si-Ca-P-based ceramic under thermal treatment. J. Eur. Ceram. Soc..

[25] Ślósarczyk A., Paluszkiewicz C., Gawlicki M., Paszkiewicz Z. (1997). The FTIR spectroscopy and QXRD studies of calcium phosphate based materials produced from the powder precursors with different CaP ratios. Ceram. Int..

[26] Mate-Sanchez de Val J.E., Mazón P., Piattelli A., Calvo-Guirado J.L., Mareque Bueno J., Granero Marín J., De Aza P.N. (2018). Comparison among the physical properties of calcium phosphate-based bone substitutes of natural or synthetic origin. Int. J. Appl. Ceram. Technol..

[27] De Aza P.N., Luklinska Z.B., Guitian F., De Aza S. (1998). Electron microscopy of interfaces in a wollastonite-tricalcium phosphate bioeutectic. J. Microsc.-Oxf..

[28] De Aza P.N., Luklinska Z.B., Mate-Sanchez de Val J.E., Calvo-Guirado J.L. (2013). Biodegradation process of α-tricalcium phosphate and α-tricalcium phosphate solid solution bioceramics in vivo: A comparative study. Microsc. Microanal..

[29] Mate-Sanchez de Val J.E., Calvo-Guirado J.L., Delgado-Ruiz R.A., Ramirez-Fernandez M.P., Negri B., Abboud M., Martinez I.M., De Aza P.N. (2012). Physical properties, mechanical behavior, and electron microscopy study of a new α-TCP block graft with silicon in an animal model. J. Biomed. Mater. Res. Part A.

[30] Townsend J.M., Dennis S.C., Whitlow J., Feng Y., Wang J., Andrews B., Nudo R.J., Detamore M.S., Berkland C.J. (2017). Colloidal gels with extracellular matrix particles and growth factors for bone regeneration in critical size rat calvarial defects. AAPS J..

[31] Hounsfield G.N. (1973). Computerized transverse axial scanning (tomography): 1. Description of system. Br. J. Radiol..

[32] Kroger H., Venesmaa P., Jurvelin J., Miettinen H., Suomalainen O., Alhava E. (1998). Bone density at the proximal femur after total hip arthroplasty. Clin. Orthop. Rel. Res..

[33] Norton M.R., Gamble C. (2001). Bone classification: An objective scale of bone density using the computerized tomography scan. Clin. Oral Implants Res..

[34] Weinberg L.A. (1993). CT scans as a radiologic database for optimum implant orientation. J. Prosthet. Dent..

[35] Aamodt A., Kvistad K.A., Andersen E., Lund-Larsen J., Eine J., Benum P., Husby O.S. (1999). Determination of the Hounsfield value for CT-based design of custom femoral stems. J. Bone Joint.

[36] Gibbons C.E., Davies A.J., Amis A.A., Olearnik H., Parker B.C., Scott J.E. (2001). Periprosthetic bone mineral density changes with femoral components of differing design philosophy. Int. Orthop..

[37] Schmidt R., Muller L., Kress A., Hirschfelder H., Aplas A., Pitto R.P. (2002). A computed tomography assessment of femoral and acetabular bone changes after total hip arthroplasty. Int. Orthop..

[38] Duckmanton N.A., Austin B.W., Lechner S.K., Klineberg I.J. (1994). Imaging for predictable maxillary implants. Int. J. Prosthodont..

[39] Jaffin R.A., Berman C.L. (1991). The excessive loss of Brånemark fixtures in type IV bone: A 5-year analysis. J. Periodontol..

[40] Pitto R.P., Mueller L.A., Reilly K., Schmidt R., Munro J. (2007). Quantitative computer-assisted osteodensitometry in total hip arthroplasty. Int. Orthop..

